# Occurrence of Pine Gall Rust on Huangshan Pine Caused by *Cronartium orientale* in China

**DOI:** 10.3390/plants15111683

**Published:** 2026-05-29

**Authors:** Shengrong Su, Qingyan Wen, Jinrong Zhu, Yao Chen, Lifeng Zhou

**Affiliations:** 1 College of Life and Environment Science, Huangshan University, Huangshan 245041, China; 107043@hsu.edu.cn (S.S.); zhongzi0531@outlook.com (Q.W.); zjr291488@outlook.com (J.Z.); 2 National Key Laboratory for Development and Utilization of Forest Food Resources, College of Forestry and Biotechnology, Zhejiang A & F University, Hangzhou 311300, China; 2023602122008@stu.zafu.edu.cn

**Keywords:** *Cronartium orientale*, pine gall rust, *Pinus taiwanensis*, alternate hosts, life cycle

## Abstract

Pine gall rust has emerged as a serious threat to *Pinus taiwanensis* (Huangshan pine) in Huangshan Mountain, a UNESCO World Heritage site in China. This study systematically investigated the etiology, host range, and epidemiological characteristics of the disease through field surveys, morphological observations, molecular phylogenetic analyses, and inoculation tests. The pathogen was identified as *Cronartium orientale* based on multi-locus sequencing (SSU, LSU, and ITS) and distinct basidiospore morphology. *Quercus stewardii* and *Castanea seguinii* were confirmed as alternate hosts, with *Q. stewardii* showing higher susceptibility. Microscopic examination revealed detailed spore morphology, and germination assays demonstrated that aeciospores and urediniospores germinate optimally at 12 °C and under near-saturated humidity. Aeciospore dispersal peaked from late April to early May, with spores detected up to 8 m from infected trees, under nearly windless conditions. The life cycle of *C. orientale* in this region involves annual production of pycnia and aecia on pine, followed by uredinia and telia on alternate hosts, enabling repeated infections. These findings clarify the etiology and epidemiology of pine gall rust on Huangshan pine, providing a scientific basis for disease monitoring and management strategies to protect the ecologically and culturally valuable Huangshan pine forests.

## 1. Introduction

*Pinus taiwanensis* Hayata is an evergreen coniferous tree that is widely distributed in open spaces and sunny ridges on sandy acidic soils of mountains with an altitude of 600–2800 meter (m) in the subtropical center of China [1]. They are tall arbor trees of 20–50 m, their trunks are straight with an umbrellalike crown, and their barks are dark gray to grayish brown. Winter buds are pinkish brown to reddish brown, cylindric or ovoid, 1–1.5 centimeter (cm) × 5–6 millimeter (mm). Needles are serrulate, 2 per bundle. Seed cones are light brown to chocolate brown, lustrous, 3–6 × 3–5 cm [2]. *P. taiwanensis* is commonly known as Huangshan pine in the Chinese Mainland and Taiwan pine in Taiwan. It is mainly found in the mountain forests of Anhui, Fujian, Guangxi, Guizhou, Henan, Hubei, Hunan, Jiangsu, Jiangxi, Taiwan, Yunnan and Zhejiang provinces [3]. It is also an important afforestation tree in the middle–lower reaches of the Yangtze River [4]. In addition, due to the natural endowment of establishing pure forests on nutrient-poor sandy soil, *P. taiwanensis* is very helpful for soil and water conservation [5]. However, in recent years, *P. taiwanensis* trees were found to have pine gall rust symptoms, which resulted in growth cessation or even death of individual trees in Huangshan Mountain (also known as Yellow Mountain). This situation could dramatically affect all the economic, ecological and landscaped benefits of *P. taiwanensis* forests.

Pine gall rust is a serious disease in pine forests of the Northern Hemisphere, causing a reduction in growth, deformity and mortality. Mortality could occur quickly in infected pine seedlings, while the infected older pine trees could survive for years [6]. In general, the highest levels of mortality occur in pine trees less than 20 years old [7]. There are two distinct types of this disease, commonly known as eastern gall rust and western gall rust [8]. Eastern gall rust, also known as pine-oak gall rust, caused by *Cronartium quercuum* ((Berk.) Miyabe ex Shirai), affects two- and three-needle pines, including Jack pine (*P. banksiana* L.), Masson pine (*P. massoniana* L.), Red pine (*P. resinosa* Ail.), Ponderosa pine (*P. ponderosa* Douglas ex Lawson), Scots pine (*P. sylvestris* L.) and other hard pines [9]. It requires pine and oak trees to complete its life cycle; alternate hosts are *Quercus* species, generally including Bur oak (*Q. macrocarpa* Michx.), Mongolian oak (*Q. mongolica* Fisch. ex Ledeb.), Pin oak (*Q. palustris* Muenchh.), and Northern red oak (*Q. rubra* L.) [10]. This disease is found throughout North and South America and Asia. The *P. densiflora* (Sieb. et Zucc.) gall rust, which had hitherto been assigned to *C. quercuum* distributed in North America, was imputed to *C. orientale* (Kaneko) in Japan in 2000 [10]. Subsequently, Zhao et al. confirmed the presence of spermagonia/spermatia and aecia/aeciospores of *C. orientale* and *C. flaccidum* (Alb. & Schwein.) on *P. hwangshanensis* (Hsia) (syn. *P. taiwanensis*) by analyzing specimens collected from Chuxian, Anhui, and another location in Anhui, China, respectively [11]. The host range of *C. orientale* also includes various other pine species, such as *P. banksiana*, *P. densata*, *P. luchuensis*, *P. mugo*, *P. nigra*, *P. pinaster*, *P. ponderosa*, *P. sylvestris*, *P. tabuliformis* var. mukdensis, and *P. thunbergii*. Its alternate hosts are *Castanea crenata*, *Castanopsis sieboldii*, and multiple species of *Quercus* spp., e.g., *Q. acutissima*, *Q. aliena*, *Q. aquifolioides*, *Q. fabri*, *Q. glauca*, *Q. liaotungensis*, *Q. mongolica*, *Q. myrsinifolia*, *Q. phellos*, *Q. rubra*, *Q. semecarpifolia*, *Q. serrata*, *Q. spinosa* and *Q. variabilis*. In contrast, western gall rust, also known as pine-pine gall rust, caused by *Peridermium harknessii* Moore (syn. *Endocronartium harknessii* (Moore) Hiratsuka), affects most two- and three-needled *Pinus* species in North America, but the disease is especially common on Lodgepole pine (*P. contorta* Dougl.) and Ponderosa pine [12]. *P. harknessii* is an autoecious fungus and needs no alternate host. It is also considered to be a serious threat to the susceptible pines grown in the Southern Hemisphere [13].

Pine gall rust has attracted increasing attention in China in recent years, owing to its discovery as an epidemic disease on Huangshan pine in Huangshan Mountain. The disease is occurring even in the most well-known monumental Huangshan pines in the Mountain, such as “King pine” (ca 500 years old) and “Queen pine” (ca 450 years old). Huangshan Mountain is located in Anhui Province, covering 1200 square kilometers, has been on the list of UNESCO world heritage site and is one of China’s 10 most popular tourist destinations (https://whc.unesco.org/en/list/547, accessed on 24 March 2026). However, the causal agent or alternate hosts of pine gall rust on Huangshan pine in Huangshan Mountain are still unclear. Therefore, the current study was conducted to characterize the pathogen causing pine gall rust on Huangshan pine on the basis of morphology and molecular analysis. Furthermore, the symptoms, conditions of pathogen development, alternate hosts and transmission characteristics were also investigated.

## 2. Results

### 2.1. The Occurrence of Pine Gall Rust on Huangshan Pine in Huangshan Mountain

**Figure 1:**
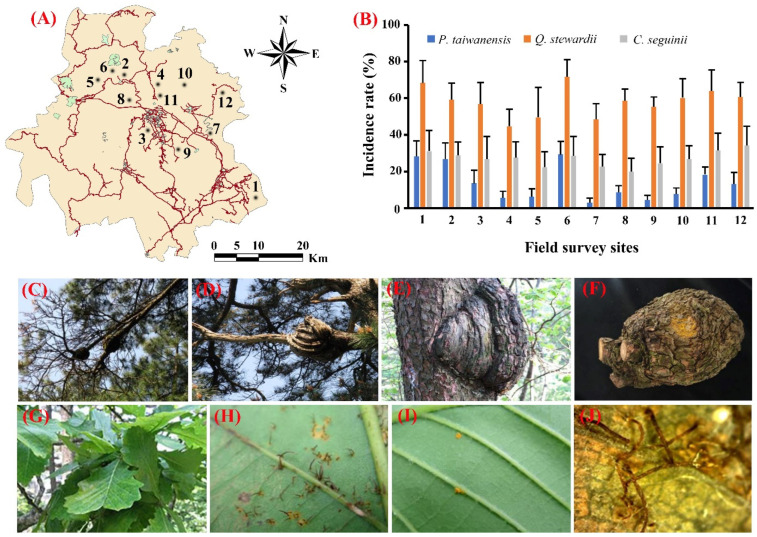
Incidence of pine gall rust on *Pinus. taiwanensis* in Huangshan Mountain. (**A**), 12 field investigation sites; 1, Cloud Valley area; 2, West Sea Grand Canyon area; 3, Baiyun Stream area; 4, King Pine area; 5, Cloud-dispelling Pavilion area; 6, Danxia Station area; 7, Bright Top area; 8, Flying-over Rock area; 9, Heaven Sea area; 10, Refreshing Terrace area; 11, White Goose Ridge area; 12, Twined Pines area. The light green areas represent lakes, and the red lines represent roads. (**B**), incidence rate of rust on *P. taiwanensis, Quercus stewardii*, and *Castanea seguinii*; data are presented as mean ± standard error. (**C**–**F**), galls of rust on *P. taiwanensis*. (**G**), adaxial surface of uredinia on *Q. stewardii* leaves. (**H**), telia on the abaxial side of *Q. stewardii* leaves. (**I**,**J**), uredinia and telia on the abaxial side of *C. seguinii* leaves.

Pine gall rust incidence rate varies across different field investigation sites, with diseased trees tending to be clustered in distribution. The Danxia Station area has the highest incidence of pine gall rust, reaching as high as 29.3%. The incidence at Cloud Valley area and West Sea Grand Canyon area is also notably high, at 28.4% and 26.8%, respectively. In contrast, King Pine area, Bright Top area and Heaven Sea area of Huangshan Mountain rarely see galls, with an incidence rate of only approx. 4% (Figure 1A,B). Galls are generally nearly spherical with varying sizes (from 5 cm to 50 cm in diameter), mainly occurring on the trunk and branches of *P. taiwanensis* (Figure 1C–E). The epidermis of the galls exhibits irregular cracking. New skin grows within the same year at the cracked areas, which will then crack again the following year. Honey-like orange droplets with spermatia ooze from the cracked areas of the gall in February, and yellow blister-like aecidia develop beneath the epidermis of the gall, emerge by rupturing the epidermis, and subsequently release the yellow powdery aeciospores in late April (Figure 1F). Uredinia and telia were found on the abaxial sides of *Q. stewardii* and *C. seguinii* leaves in all investigated sites, and the disease incidence of *Q. stewardii* was recorded between 44.8% and 71.6%, markedly exceeding that of *C. seguinii* (between 20.1% and 34.3%) (Figure 1B). From mid-May to early July, distinct bright yellow spots (uredinia) appear on the abaxial sides of *Q. stewardii* and *C. seguinii* leaves, while the corresponding adaxial leaf surfaces exhibit slightly lighter coloration compared to healthy areas (Figure 1G,I). From early July to late September, hair-like structures (telia) emerge from the uredinium, with each uredinium producing one to more than ten telia. Initially, telia are light brown, they gradually turn reddish brown and finally dark brown, and often they are slightly curved (Figure 1H,J).

### 2.2. DNA-Based Identification of Pine Gall Rust on Huangshan Pine

**Figure 2:**
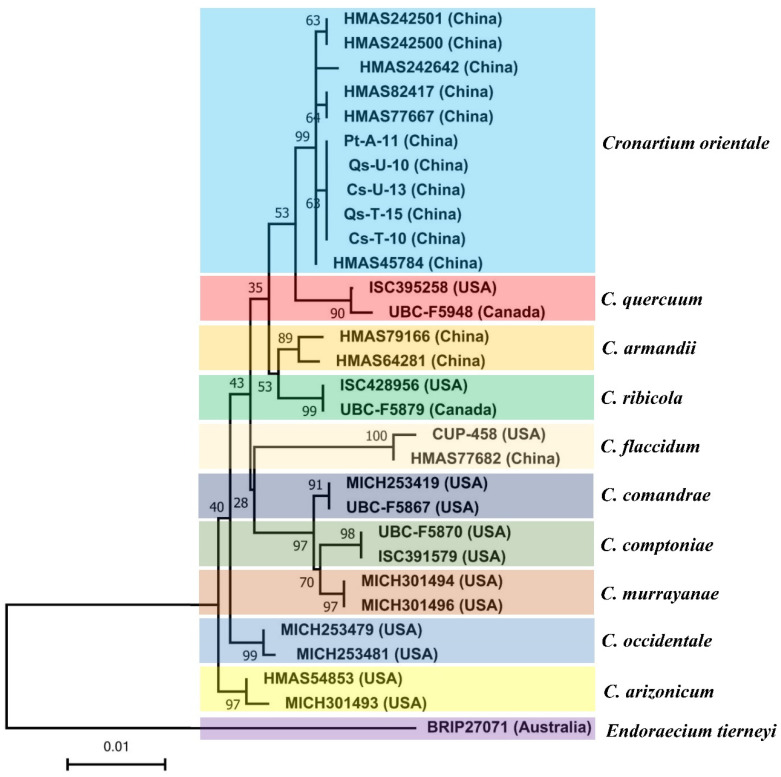
Phylogenetic relationships of *Cronartium* species based on SSU and LSU sequences. Bootstrap values from 1000 replicates are shown above the branches for the neighbour-joining tree. Scale bar indicates the distance caused by 10 nucleotide substitutions. Pt-A-11, Qs-U-10, Cs-U-13, Qs-T-15 and Cs-T-10 were obtained in this study.

**Figure 3:**
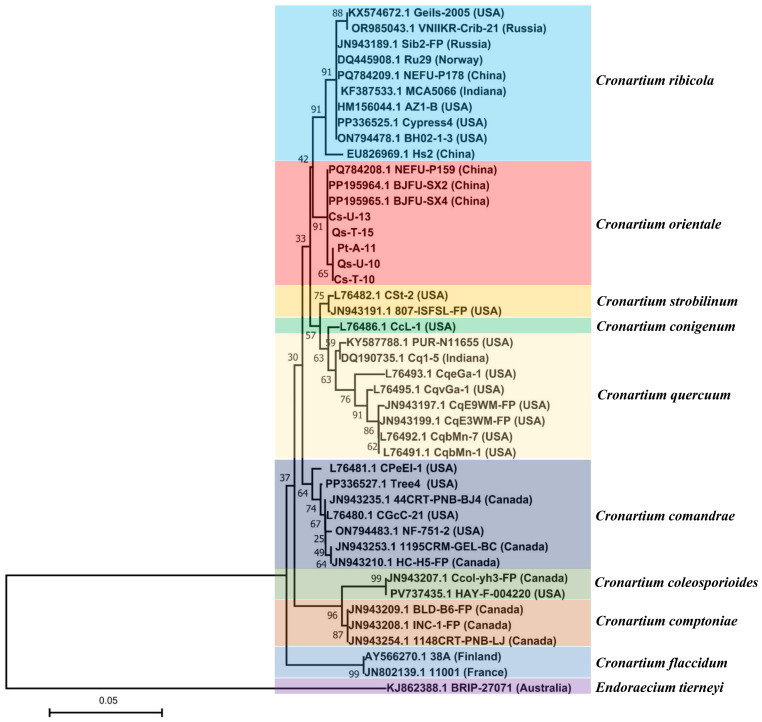
Phylogenetic relationships of *Cronartium* species based on ITS sequences. Bootstrap values from 1000 replicates are shown above the branches for the neighbour-joining tree. Scale bar indicates the distance caused by 50 nucleotide substitutions. Pt-A-11, Qs-U-10, Cs-U-13, Qs-T-15 and Cs-T-10 were obtained in this study.

The PCR products of the three nuclear ribosomal RNA gene regions obtained from different spores in the three host tree species were submitted to the GenBank (Table 1 and Table 2), and then aligned with the previously published sequences by Basic Local Alignment Search Tool (https://blast.ncbi.nlm.nih.gov/Blast.cgi, accessed on 21 July 2025). Because there are quite a few strains of *Cronartium* spp. with incomplete sequence information of the ITS, multigene joint tree construction was performed with SSU and LSU regions. The resulting maximum likelihood tree was topologically congruent with the neighbor-joining methods tree. The phylogenetic relationship showed that the newly obtained sequences clustered with *C. orientale*, forming a separate clade distinct from other species in the *Cronartium* genus in the Neighbour-joining method tree (Figure 2). The clade containing *C. orientale* and our sequences received high bootstrap support (≥99%), and the genetic distance between our isolates and the reference *C. orientale* was very low (e.g., 0.1–0.2% in the combined SSU + LSU regions), whereas distances to other species such as *C. quercuum* and *C. ribicola* exceeded 0.6%. These results confirm the distinct species boundary. Phylogenetic trees containing complete ITS sequences of other strains (Table 2) were constructed to further identify newly obtained sequences, and the result (Figure 3) was in accord with the above multigene joint tree. Therefore, the molecular phylogenetic analysis indicated that *C. orientale* is the causative agent of pine gall rust on Huangshan pine; *Q. stewardii* and *C. seguinii* are very likely to be the alternate hosts of the rust.

### 2.3. Alternate Hosts of Pine Gall Rust on Huangshan Pine

Approximately two weeks post-inoculation, uredinia developed on the leaves of *Q. stewardii* and *C. seguinii* seedlings. During the recording period, all of the 30 inoculated seedlings (15 seedlings of each species) appeared to show symptoms similar to those of the naturally infected trees in the field (Figure 1G–J). None of the seedlings in the control group exhibited any symptoms. Uredinia of *C. orientale* was re-isolated from infected leaves of the inoculated seedlings and identified as previously described, thus fulfilling Koch’s postulates [14]. These results further indicate that *Q. stewardii* and *C. seguinii* are the alternate hosts of pine gall rust on Huangshan pine in Huangshan Mountain.

### 2.4. Microscopic Characteristics of C. orientale

**Figure 4:**
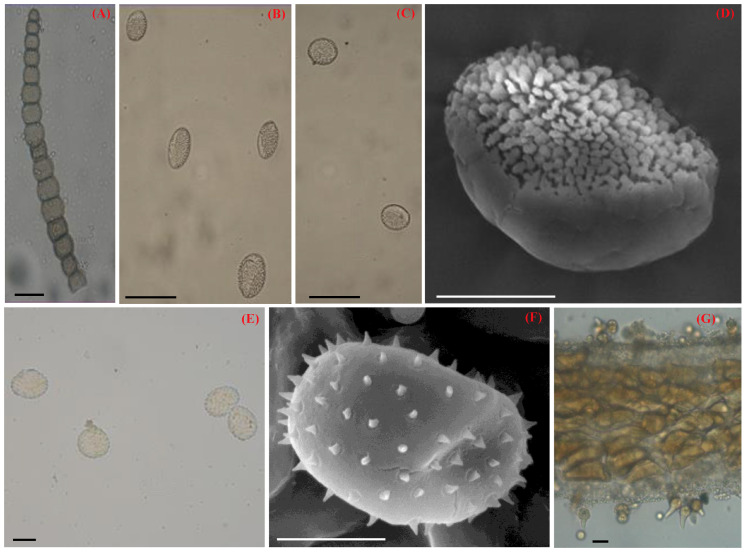
Microscopic characteristics of *Cronartium orientale* on Huangshan pine. (**A**), concatenate of aeciospores; (**B**,**C**), globose and ellipsoidal aeciospores; (**D**), scanning electron micrograph of aeciospore; (**E**), ovoid uredospore; (**F**), scanning electron micrograph of uredospore; (**G**), spherical basidiospore with a rostrate projection. Scale bar: (**A**) = 10 μm, (**B**,**C**) = 50 μm, (**D**) = 10 μm, (**E**) = 15 μm, (**F**) = 5 μm, (**G**) = 15 μm.

The aeciospores are concatenate (Figure 4A), appearing orange-yellow in mass, while individually they are bright yellow, globose to ellipsoid (Figure 4B,C), measuring 24.1–36.2 × 16.1–23.5 μm (*n* = 50). Under scanning electron microscope, the surface of the aeciospores exhibits verrucose regions consisting of cylindrical warts, each with 3–4 annular ridges. The remaining areas are smooth and display a reticulate pattern (Figure 4D). Urediniospores are bright yellow and ovoid, with echinulate walls (Figure 4E,F), 15.9–24.8 × 12.7–19.3 μm (*n* = 50). Basidiospores are spherical, hyaline, and smooth, with a rostrate projection (Figure 4G), 10.1–12.2 μm (*n* = 50) in diameter.

### 2.5. Germination Characteristics of Aeciospore and Uredospore

**Figure 5:**
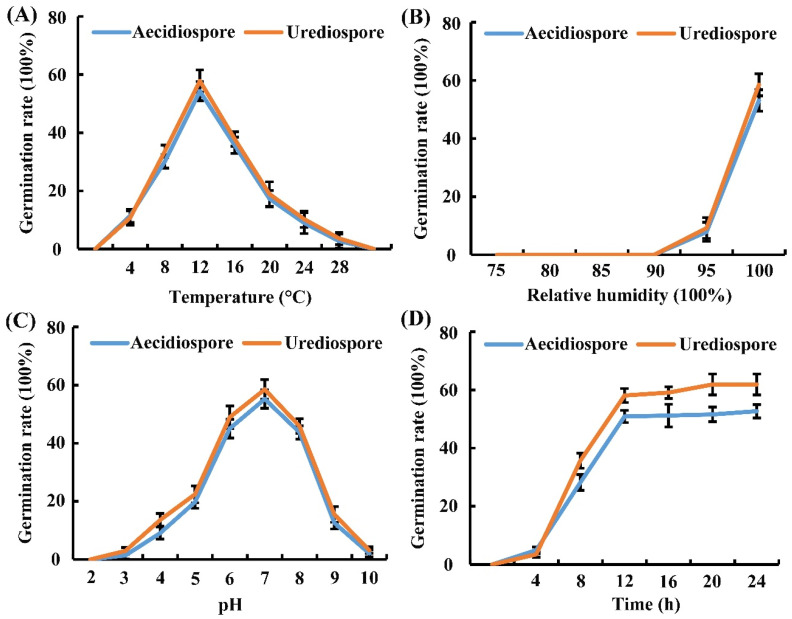
Germination characteristics of aecidiospores and urediniospores. (**A**), temperature ranges for germination of aecidiospores and urediniospores; (**B**), relative humidity ranges for germination of aecidiospores and urediniospores; (**C**), pH ranges for germination of aecidiospores and urediniospores; (**D**), time ranges for germination of aecidiospores and urediniospores. Bars represent mean ± standard error.

The aecidiospores and urediniospores of *C. orientale* were capable of germination within a temperature range of 4 to 28 °C. The optimal temperature range for germination was 8 to 16 °C, with the most favorable temperature being 12 °C. Germination rates decreased sharply when temperatures exceeded 20 °C (Figure 5A). Germination of aecidiospores and urediniospores of *C. orientale* was strictly dependent on humidity, occurring optimally only under conditions of extremely high humidity (approaching 100%). Even at 90% relative humidity, the germination rate was extremely low, to the point of being virtually negligible (Figure 5B). Aecidiospores and urediniospores of *C. orientale* were capable of germination across a pH range of 3 to 10, with an optimum at pH 7. At pH 7, the germination rates recorded for aecidiospores and urediniospores were 55.21% and 58.46%, respectively (Figure 5C). The primary germination period for both aecidiospores and urediniospores of *C. orientale* occurred between 8 and 12 h of incubation. By 12 h, the majority of aecidiospores and urediniospores had already germinated, with the germination rates reaching their peak at 20 h (Figure 5D).

### 2.6. Transmission Characteristics of Aecidiospores

**Figure 6:**
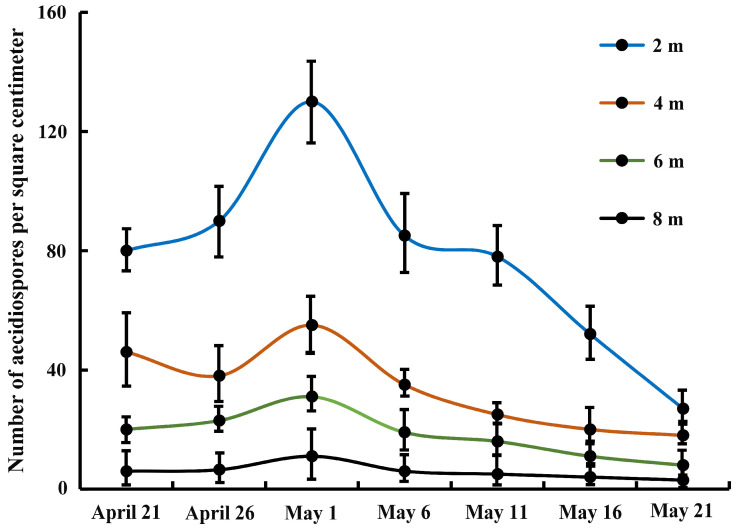
Transmission characteristics of aecidiospores in the galls on Huangshan pines. 2 m, 4 m, 6 m, and 8 m represent the respective distances of the aecidiospore collectors (coverslips (1 cm × 1 cm) covered with vaseline) from the trunk of pine trees with galls. Bars represent mean ± standard error.

The highest number of aeciospores was collected by the coverslips with vaseline at the distance of 2 m from the trunk under the pine trees with galls, with progressively fewer aeciospores collected at the distance of 4 m and 6 m from the trunk. Aeciospores could be detected at distances of up to 8 m from the trunks (Figure 6). These aeciospores were commonly observed in clusters, which may be attributed to their catenulate formation. The collection of aeciospores peaked in late April and early May, with hundreds of aeciospores per coverslip collected under the pine trees with galls. This indicates that this period represents the peak time for aeciospore dispersal.

### 2.7. Life Cycle of Pine Gall Rust on Huangshan Pine

**Figure 7:**
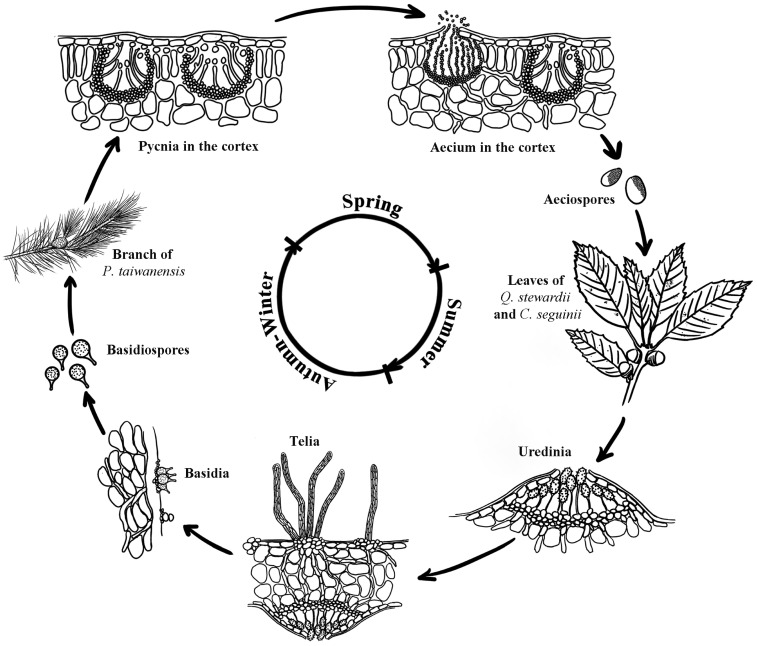
Life cycle of pine gall rust on Huangshan pine in Huangshan Mountain.

Our field surveys revealed that galls on the Huangshan pines produced pycnia containing pycniospores within the cortex annually from January to February. In late April, after fertilization, aecia developed in the tissues beneath the pycnia. The aeciospores were dispersed by wind to the leaves of *Q. stewardii* and *C. seguinii* in the mountain regions, where they germinated under cool and humid conditions. From May to June, uredinia were formed, and the resulting urediniospores repeatedly infected *Q. stewardii* and *C. seguinii*. From July to September, these uredinial sori were progressively replaced by telia (the teliospore-producing structures). After the teliospores matured (mid-August to late September), they germinated to produce basidia (promycelia) bearing basidiospores when external temperatures were suitable. These basidiospores were dispersed by wind to Huangshan pines (Figure 7). Once the galls were established on Huangshan pines, they annually produced aecia beneath the cortex, which then released aeciospores that were dispersed by wind to the leaves of *Q. stewardii* and *C. seguinii*. This cycle repeated, and the galls on Huangshan pines enlarged progressively each year.

## 3. Discussion

In the past, the pathogen responsible for the widespread pine gall rust in East Asia was consistently identified as its “relative,” the pine-oak gall rust fungus *C. quercuum*, which is distributed in North America [15]. In recent years, with advancements in taxonomic techniques, particularly the application of morphological comparisons and DNA sequencing analysis, researchers have discovered significant and stable differences between the Asian and North American populations [16,17]. Morphologically, the key to distinguishing *C. orientale* from its close relative *C. quercuum* lies in the shape of the basidiospores [10,18]. The basidiospores of *C. orientale* are globose and appear almost transparent (nearly colorless) under microscopic observation. In contrast, the basidiospores of *C. quercuum* are ellipsoid and exhibit a characteristic yellow-orange color [10]. This aligns with the results we have observed regarding basidiospore morphology in the case of pine gall rust on *P. taiwanensis*. Furthermore, in terms of molecular systematics, *C. orientale* and *C. quercuum* fell into two well-supported, independent clades.

Uredinia and telia of pine gall rust were found on the abaxial sides of *Q. stewardii* and *C. seguinii* leaves in all investigated sites, but the incidence rate of *Q. stewardii* was substantially higher than that of *C. seguinii*. It indicated that *Q. stewardii* exhibits greater susceptibility to *C. orientale* than *C. seguinii*. On the abaxial leaf surfaces, *Q. stewardii* is characterized by stellate trichomes flanking the midrib, while *C. seguinii* exhibits a dense covering of peltate (scalelike) glands [19], a structural distinction that could influence spore attachment of *C. orientale*. This might explain the difference in susceptibility of the two species to *C. orientale*. The period from late April to early May marks the peak maturation of aeciospores of pine gall rust. During this time, the alternate hosts, *Q. stewardii* and *C. seguinii*, are in the stage of leaf expansion [20,21]. The newly matured aeciospores are dispersed by wind onto the freshly unfolded leaves of these alternate hosts. This might perhaps represent a form of “synchronization” achieved through long-term coevolution between the pathogen and its hosts [22], potentially involving volatile signaling compounds, a hypothesis that warrants further in-depth investigation.

The aeciospores and urediniospores of the pine gall rust on Huangshan pine trees are sensitive to high temperatures, with an optimal germination temperature range of 8–16 °C. When the temperature exceeds 20 °C, the germination rate drops sharply. Additionally, the germination of these spores is closely related to humidity; they can only germinate well under extremely high-humidity conditions (approximately 100% relative humidity), and even at 90% relative humidity, the germination rate is extremely low, making germination nearly impossible. It is highly likely that it is precisely this close relationship with climatic factors that causes the outbreak of pine gall rust on *P. taiwanensis* in Huangshan Mountain. During the period from May to August, when the aeciospore and urediniospore stages of the rust fungus develop, the monthly average temperature in the Huangshan Scenic Area (600–1800 m elevation) ranges from 8 to 19 °C, accompanied by abundant precipitation (monthly rainfall of 150–550 mm) [23,24]. These conditions coincide precisely with the requirements for aeciospore and urediniospore germination of the pine gall rust. Pine gall rust on *P. taiwanensis* occurs mainly in the Huangshan Scenic Area at elevations between 600 and 1800 m, whereas in forest stands below 600 m, although both host species (pine and oak) are present, the disease is rarely observed. Therefore, it is suggested that the outbreak of pine gall rust in the Huangshan Scenic Area may persist and continue to affect the health of *P. taiwanensis*.

The strong dependence of aeciospore and urediniospore germination on low temperatures and near-saturated humidity provides a clear basis for managing pine gall rust in the Huangshan Scenic Area. First, silvicultural practices should aim to reduce within-stand humidity and break the microclimatic optimum for spore germination. Selective thinning to improve air circulation, pruning of lower branches, and avoiding dense regeneration in high-risk elevations could lower relative humidity below the threshold required for infection. Second, early detection strategies should target the critical May–August window when monthly mean temperatures and heavy rainfall align with spore germination requirements. Geospatial mapping of high-risk zones (600–1800 m), combined with periodic inspection of gall development and spore formation, would enable timely application of fungicides or targeted removal of infected galls. Additionally, monitoring alternate hosts is critical. Although both pine and oak co-exist below 600 m, disease occurrence is rare there, suggesting that transmission is elevation-dependent. Regular surveys of oak species in the 600–1800 m zone during May–August could identify inoculum sources before spring spore dispersal. Together, these measures could mitigate the ongoing outbreak and protect the health of *P. taiwanensis* in the Huangshan Scenic Area.

## 4. Materials and Methods

### 4.1. Field Investigation

Field investigations were conducted from January 2018 to December 2020 in Huangshan Mountain at 12 sites, between 118°01′ and 118°17′ east longitude and between 30°01′ and 30°18′ north latitude, with an altitude of 400–1800 m. Galls were cut from branches or trunks of Huangshan pines and transported to laboratory for collection of aecidiospores in the 12 investigated sites. The ecological environment and surrounding vegetation conditions of pine forest was investigated. Uredinia and telia were also found on the leaves of *Q. stewardii* and *C. seguinii* near the diseased pine trees in all investigated sites. Samples from the two tree species were also collected and transported to laboratory for examination of uredinia and telia. Incidence rate (%) = number of trees with gall (or uredinia)/total number of investigated trees × 100%, which was introduced to indicate the degree of the disease. At each of the 12 investigated sites, 30 randomly selected Huangshan pines were examined for the presence of galls. For the other two species, *Q. stewardii* and *C. seguinii*, 20 individuals of each species were randomly chosen per site. The sampling strategy followed a randomized design to avoid bias: within each site, trees were numbered and a random number table was used to select the target trees. For each selected tree, we collected gall samples from the main branches or trunk, and leaves showing uredinia or telia from *Q. stewardii* and *C. seguinii*.

### 4.2. Molecular Phylogenetic Analysis

As uredinia and telia were discovered on the abundant leaves of *Q. stewardii* and *C. seguinii* near the diseased pine trees, the two species were supposed to be alternate hosts of pine gall rust. For genetic analysis, we collected aeciospores from the galls of *P. taiwanensis*, as well as uredinia and telia from the leaves of *Q. stewardii* and *C. seguinii*, across all 12 investigation sites. Genomic DNA was extracted from the above-mentioned samples using the Master Pure Yeast DNA Purification Set Kit (Sigma, Rehovot, Israel) according to the manufacturer’s instructions. Then, the DNA was quantified using a NanoDrop spectrophotometer ND-1000 (Gene Company Ltd., Hong Kong, China). Three nuclear ribosomal RNA gene regions, small subunit (SSU) rDNA, internal transcribed spacer regions (ITS) and large subunit (LSU) rDNA, were employed for phylogenetic analysis [11]. PCR and sequencing of the three gene regions were performed with primer sets NS1/Rust18SR, ITS5-u/ITS4rust and LRust1R/LR6 [25]. One representative sample per host and per sampling site was sequenced. The reaction conditions were as follows: initial denaturation at 95 °C for 5 min; then 30 cycles of denaturation at 95 °C for 30 s, annealing at temperatures ranging from 55 °C to 65 °C for 30 s, and extension at 72 °C for 60 s; followed by a final extension at 72 °C for 10 min. The newly obtained sequences of the three gene regions were aligned with the corresponding genes of related species (Table 1 and Table 2), and phylogenetic trees were constructed using both maximum likelihood and Neighbor-joining method with 1000 bootstrap replications using PhyloSuite v1.2.2 [26] and MEGA X software Version 7 [27]. *Endoraeciu tierneyi* was selected as outgroup.

### 4.3. Potential Alternate Hosts Analysis

For further clarity on alternate hosts of the pine gall rust on Huangshan pine in Huangshan Mountain, inoculation tests were carried out in the greenhouse of Huangshan University in 2019. Aecidiospores collected from galls of Huangshan pines were transported to Petri dishes in mid-May. *Q. stewardii* and *C. seguinii* were inoculated by dusting aecidiospores on the two-year-old seedling leaves [28,29]. Prior to inoculation, the leaves of the seedlings were moistened using a water sprayer, and after inoculation, the seedlings were incubated in the greenhouse separately and misted for 3 s every 30 min for 10 weeks to maintain humidity. The greenhouse was maintained at 14 °C (the average temperature in the Huangshan Scenic Area from May to June) with a natural photoperiod of 14 h light and 10 h dark. Fifteen seedlings of each species were inoculated, and an equal number of seedlings were mock-inoculated with sterilized water as a control. Incidence rates of *Q. stewardii* and *C. seguinii* seedlings were recorded over 10 weeks from inoculation as uredinia developed from the leaves.

### 4.4. Microscopic Observation

Galls cut from Huangshan pines were transported to laboratory for morphologic observation of aecidiospores in late April 2019. Detailed examination to identify the aecidiospores was conducted using a Zeiss Axio Image M2 microscope (Zeiss MicroImaging GmbH, Oberkochen, Germany) and a scanning electron microscope (SEM) to take photographs [30]. Fixed specimens were processed for scanning electron microscope according to the following steps: samples were washed using 0.1 M phosphate buffer (pH 7.2) and fixed with 2.5% glutaraldehyde; thereafter, the samples were dehydrated, freeze-dried, and sputter-coated with gold-palladium. Uredinia collected from the leaves of *Q. stewardii* and *C. seguinii* were brought to laboratory for morphologic observation of urediniospores in late May 2019. Detailed examination to identify the urediniospores was conducted using the same approach as above. In July 2019, telia collected from the leaves of *Q. stewardii* and *C. seguinii* were brought to laboratory for morphologic observation of teleutospores and basidiospores.

### 4.5. Assay Aecidiospore and Urediniospore Germination Rates

For clarity on the germination characteristics of aecidiospore and urediniospore, four different tests were carried out, respectively. Aecidiospores and urediniosporeswere collected from the field as described above, suspended, and the concentration was adjusted to 10^4^/mL, respectively. I. Effect of different temperature on aecidiospore and urediniospore germination: a circle of absorbent paper was folded to fit a 9 cm dish, placed in the dish and soaked in water. Aliquots of aecidiospore and urediniospore suspensions were then applied to concave glass slides, which were placed in the dish, respectively. The petri dishes were kept in the constant temperature incubators (TR-R420C, Zhejiang Top Yunnong Technology Co., Ltd., Hangzhou, China) of 0 °C, 4 °C, 8 °C, 12 °C, 16 °C, 20 °C, 24 °C and 28 °C for 12 h, respectively. Then, germination rate of 100 randomly selected aecidiospores or urediniospores in each slide was recorded under a light microscope. II. Effect of relative humidity (RH) on aecidiospore and urediniospore germination: suspensions of aecidiospores and urediniospores were incubated in the constant humidity incubators (TR-R420C) of 75%, 80%, 85%, 90% and 95% at 12 °C for 12 h, respectively. The relative humidity of sterilized H_2_O is taken as 100%. Then, the germination rate was recorded as described above. III. Effect of different pH conditions on aecidiospore and urediniospore germination: preparing different values of pH solutions, 2.0, 3.0, 4.0, 5.0, 6.0, 7.0, 8.0, 9.0 and 10.0, using 0.1 mol/L HCl and NaOH solutions. Aliquots of aecidiospore and urediniospore suspensions were applied to the above different pH solutions at 12 °C for 12 h. The germination rate was recorded as described above. IV. The time required for aecidiospore and urediniospore germination: Aliquots of aecidiospore and urediniospore suspensions were applied to concave glass slides, incubated in the constant temperature incubators for 4 h, 8 h, 12 h, 16 h, 20 h and 24 h at 12 °C, respectively. Then the germination rate was recorded as described above. Each treatment had five independent replicates.

### 4.6. Assay Transmission Characteristics of Aecidiospores

To investigate the transmission characteristics of pine gall rust on Huangshan pine, the number of aecidiospores was assessed in the forest from early April in 2019. Five Huangshan pine trees (approx. 10 m above the ground) bearing galls (approx. 7 m above the ground) in the almost windless mountain coves were selected as replicates. For each tree, coverslips (1 cm × 1 cm) covered with vaseline were placed under the pine trees with galls at a distance of 2 m, 4 m, 6 m and 8 m in four cardinal directions of each tree for 24 h. Then, aecidiospores in each coverslip were observed under a light microscope and recorded. The assessments were conducted every five days with five replicates.

## Figures and Tables

**Table 1:** Species of *Cronartium* and GenBank accession numbers of sequences (SSU and LSU) used for phylogenetic studies. Accession numbers in bold were obtained in this study.

Species	Specimen No.	Host	Country	GenBank Accession No.	
				SSU	LSU
*C. arizonicum*	HMAS54853	*Pinus ponderosa*	USA	OM745899	OM746341
	MICH301493	*P. ponderosa*	USA	OM745901	OM746343
*C. armandii*	HMAS79166	*Ribes maximowiczii*	China	OM745904	MZ520623
	HMAS64281	*R. orientale*	China	OM745905	OM746345
*C. comandrae*	MICH253419	*Comandra* sp.	USA	OM745936	OM746372
	UBC-F-5867	*Com. pallida*	USA	OM745934	OM746370
*C. comptoniae*	UBC-F-5870	*Comptonia asplenitolia*	USA	OM745942	OM746378
	ISC391579	*Co. peregrina*	USA	OM745944	OM746380
*C. flaccidum*	CUP-458	*P. silvestris*	USA	OM745973	OM746405
	HMAS77682	*P. massoniana*	China	OM745970	OM746404
*C. murrayanae*	MICH301494	*P. murrayana*	USA	OM745993	OM746425
	MICH301496	*P. murrayana*	USA	OM745994	OM746426
*C. occidentale*	MICH253479	*R. gandfalii*	USA	OM745997	OM746429
	MICH253481	*R. aureum*	USA	OM745999	OM746431
*C. orientale*	HMAS82417	*Q. mongolica*	China	OM746006	OM746437
	HMAS77667	*Q. liaotungensis*	China	OM746005	OM746436
	HMAS242501	*Q. variabilis*	China	OM746009	OM746439
	HMAS242500	*Q. variabilis*	China	OM746008	OM746438
	HMAS242642	*Q. aquifolioides*	China	OM746002	OM746433
	HMAS45784	*P. densata*	China	OM746010	OM746440
	**Pt-A-11**	***P. taiwanen*** ***is***	**China**	**PZ246144**	**PZ246138**
	**Qs-U-10**	***Q. stewardii***	**China**	**PZ246145**	**PZ246139**
	**Cs-U-13**	***C. seguinii***	**China**	**PZ246146**	**PZ246140**
	**Qs-T-15**	***Q. stewardii***	**China**	**PZ246147**	**PZ246141**
	**Cs-T-10**	***C. seguinii***	**China**	**PZ246148**	**PZ246142**
*C. quercuum*	ISC395258	*Q. imbricaria*	USA	OM746033	OM746461
	UBC-F5948	*P. sylvestris*	Canada	OM746035	OM746463
*C. ribicola*	ISC428956	*R. missouriense*	USA	OM746057	OM746485
	UBC-F5879	*P. monticola*	Canada	OM746064	OM746492
*Endoraeciu tierneyi*	BRIP-27071	*Acacia harpophylla*	Australia	NG_065052	NG_059235

**Table 2:** Species of *Cronartium* and ITS GenBank accession numbers of sequences used for phylogenetic studies. Accession numbers in bold were obtained in this study.

Species	Isolate No.	Country	ITS GenBank Accession No.
*C. coleosporioides*	Ccol-yh3-FP	Canada	JN943207
	HAY-F-004220	USA	PV737435
*C. comandrae*	1195CRM-GEL -BC	Canada	JN943253
	HC-H5-FP	Canada	JN943210
	NF-751-2	USA	ON794483
	CPeEl-1	USA	L76481
	Tree 4	USA	PP336527
	44CRT-PNB-BJ4	Canada	JN943235
	CGcC-21	USA	L76480
*C. comptoniae*	BLD-B6-FP	Canada	JN943209
	INC-1-FP	Canada	JN943208
	1148CRT-PNB-LJ	Canada	JN943254
*C. conigenum*	CcL-1	USA	L76486
*C. flaccidum*	38A	Finland	AY566270
	11001	France	JN802139
*C. orientale*	NEFU-P159	China	PQ784208
	BJFU-SX2	China	PP195964
	BJFU-SX4	China	PP195965
	**Pt-A-11**	**China**	**PZ253841**
	**Qs-U-10**	**China**	**PZ253842**
	**Cs-U-13**	**China**	**PZ253843**
	**Qs-T-15**	**China**	**PZ253844**
	**Cs-T-10**	**China**	**PZ253845**
*C. ribicola*	Cypress-4	USA	PP336525
	Geils-2005	USA	KX574672
	Hs2	China	EU826969
	VNIIKR-Crib21	Russia	OR985043
	AZ1-B	USA	HM156044
	MCA5066	Indiana	KF387533
	NEFU-P178	China	PQ784209
	Ru29	Norway	DQ445908
	BH02-1-3	USA	ON794478
	Sib2-FP	Russia	JN943189
*C. strobilinum*	CSt-2	USA	L76482
	807-ISFSL-FP	USA	JN943191
*E. tierneyi*	BRIP-27071	Australia	KJ862388

## Data Availability

The original contributions presented in this study are included in the article. Further inquiries can be directed to the corresponding author.
